# Supplementary material for: Combination of Niraparib, Cisplatin and Twist Knockdown in Cisplatin-Resistant Ovarian Cancer Cells Potentially Enhances Synthetic Lethality through ER-Stress Mediated Mitochondrial Apoptosis Pathway
Source: Int J Mol Sci. 2021 Apr 10;22(8):3916. doi: 10.3390/ijms22083916 (PMC8070209; doi:10.3390/ijms22083916)
Supplement: Supplementary file 1 [file ijms-22-03916-s001.pdf]

Supporting Materials

1. Supplementary materials and methods

1.1. Generation of three dimensional (3D) spheroidal model

1.2 % poly-HEMA gel was prepared in 95% ethanol and coated on the bottom of 12, 24, 48 and 96-well culture plates by 500, 300, 150 and 75 μl, respectively. Then the culture plates were kept under laminar flow cabinet for air drying. The cells were seeded at 2 × 10^6 cells/ml in the form of 20 μl drops on the inverted lid of the 60 mm petri dish, while the dish contained 4 to 5 ml of PBS. The generated cell aggregate sheets were transferred to Poly-HEMA coated plate containing complete medium after 48 h. After 24 h, the spheroids derived from siNC and siTwist cells were cultured in the absence or presence of Cis, Nira or Cis+Nira for 120 h with routine checking under light microscopy. The cultures were placed at 37˚C in a humidified incubator under 5% CO_2 atmosphere throughout the culture period.

1.2. Mitochondrial apoptosis staining

After the treatment period, the 3D organoid cell suspension were collected to a centrifuge tube and centrifugation for 5 min at room temperature at 400 ×g, then remove supernatant, followed by addition of mitostain solution and incubate at 37˚C incubator for 15 min. After incubation, centrifuged for 5 min at 400 ×g, remove supernatant, then washed with PBS. Subsequently, PBS was used to mount the chamber for imaging by fluorescence microscopy and resuspended for fluorescence ratio detection by microplate reader. In fluorescence microscopy, mitostain was imaged using an Ex/Em: 540/570 nm filter, whereas the green signal was measured at Ex/Em: 490/520 nm. The fluorescence ratio was detected by measuring red fluorescence (Ex/Em: 550/600 nm) and green fluorescence (Ex/Em: 485/535) using a fluorescence microplate reader.

1.3. Mitochondrial and cytosolic separation

After treatment period, cells were washed twice with cold PBS and treated with lysis buffer containing protease inhibitors. Then they were homogenized by gentle douncing (100 strokes) and keep on ice for 30 min. Then collected the buffer and centrifuged for 10 min at 750 ×g at 4°C, and the sediment containing the nuclei and unbroken cells was discarded. The supernatant was then centrifuged at 10,000 ×g for 10 min. The resulting supernatant was removed and used as the cytosolic fraction. The sediment containing the mitochondria was further incubated with PBS containing 0.5% Triton X-100 for 10 min at 4°C. After centrifugation at 10,000 ×g for 30 min, the supernatant was collected as the mitochondrial fraction.

1.4. Western blotting

Total protein for western blotting was extracted from cells cultured in 2D and 3D environment using Pro-Prep protein extraction solution (#17081, iNtRON Biotechnology, Seongnam, Korea) including protease inhibitor cocktail (#P8340, Sigma-Aldrich, St. Louis, MO, USA), phosphotase inhibitor cocktail 2 (#P5726, Sigma-Aldrich, St. Louis, USA) and phosphotase inhibitor cocktail 3 (#P0044, Sigma-Aldrich, St. Louis, MO, USA), centrifuged at 11,000 ×g for 15 min, and quantified using a bicinchoninic acid (BCA) assay kit (#21071, iNtRON Biotechnology, Seongnam, Korea). The proteins were separated by 8 to 15% using sodium dodecyl sulfate-polyacrylamide (SDS-PAGE) gel electrophoresis (SDS-PAGE) gel and transferred to 0.2 μM polyvinylidene difluoride (PVDF)

membranes (#IB24001, Invitrogen; Thermo Fisher, Carlsbad, CA, USA) using the iBlot 2 dry blotting system (Invitrogen; Life Technology; Carlsbad, CA, USA). The primary and corresponding secondary antibodies were incubated in the iBind Western Device (Thermo Fisher Scientific, Carlsbad, CA, USA) using iBind solution kits (#INV-SLF1020, Thermo Fisher Scientific, Carlsbad, CA, USA). The primary antibodies were: mouse monoclonal anti-XRCC1 (#MA5-12071, Thermo Fisher Scientific, Carlsbad, CA, USA), mouse monoclonal anti-PARP-1 (#SC-8007, Santa Cruz Biotechnology, Dallas, TX, USA), rat monoclonal anti-GRP78 (#SC-13539, Santa Cruz Biotechnology), mouse monoclonal anti-calnexin, (#SC-23954, Santa Cruz Biotechnology), mouse monoclonal anti-ATF-6α, (#SC-16659, Santa Cruz Biotechnology), mouse monoclonal anti-CHOP (#2895, Cell Signaling Technology. Danvers, MA, USA), mouse monoclonal anti-Bax (#SC-20067, Santa Cruz Biotechnology), mouse monoclonal anti-Bcl-2 (#SC-509, Santa Cruz Biotechnology), rabbit anti-cleaved caspase-9 (ab2324, Abcam, Cambridge, MA, USA), rabbit polyclonal anti-cleaved caspase-3 (#9661, Cell Signaling Technology, Danvers, MA, USA), mouse monoclonal ant-cytochrome *c* (#SC-13156, Santa Cruz Biotechnology), mouse monoclonal anti-VDAC1 (#SC-390996, Santa Cruz Biotechnology), mouse monoclonal anti-Twist (#SC-8426, Santa Cruz Biotechnology), mouse monoclonal anti-vimentin (#SC-8426, Santa Cruz Biotechnology) and mouse monoclonal anti-β-actin (#A5441, Sigma-Aldrich, St. Louis, MO, USA). The proteins were visualized using EZ-Western Lumi Plus solution (#WSE-7120L, ATTO Corporation, Tokyo, Japan) in EZ-Capture ST (ATTO Corporation, Tokyo, Japan), and bands were measured using ImageJ software.

1.5. Histology and immunohistochemistry

The After the treatment of the 3D spheroids with DMSO, Cis alone, Nira alone and Cis+Nira combination, aspirated off the medium from each well and wash the spheroid by adding PBS for 2×5 mins, fixed them by adding 10 % formalin. Following paraffin embedding and casting into a block, 3 μm thick sections were cut and mounted onto slides. All slides were kept at 58°C for 60 min in a drying chamber, deparaffinized in xylene (#534056, histological grade, Sigma-aldrich, St. Louis, MO, USA) followed by rehydration in 100%, 95% and 70% ethanol (#A405P-4, histological grade, Fisher scientific, Hampton, New Hampshire, USA) and distilled water. Subsequent slides were stained with hematoxylin and eosin (H&E) according to routine protocols or subjected to immunohistochemistry (IHC). For IHC, the sections were placed in IHC-Tek Epitope Retrieval Solution (#IW-1100, IHC world, Woodstock, MD, USA) and then placed in IHC-Tek Epitope Retrieval Steamer Set (#IW-1102, IHC world, Woodstock, MD, USA) for 20 min to utilize steaming method to achieve antigen unmasking on formalin fixed paraffin embedded organoid sections. The sections were rinsed with IHC-Tek Washing Buffer (#IW-1201, IHC world, Woodstock, MD, USA) for 2×5 min. They were then blocked with IHC-Tek Peroxidase Blocking Solution (#IW-1300, IHC world, Woodstock, MD, USA) for 10 min and then rinsed sections with IHC-Tek Washing Buffer for 2x5 min. Incubated the sections in primary antibody at appropriate dilution in IHC-Tek Antibody Diluent (#IW-1001, IHC world, Woodstock, MD, USA). IHC was performed using antibodies for Ki-67 (anti-rabbit, 1:250, MyBioSource, San Diego, CA, USA) and cleaved caspase-3 (anti-mouse, 1:300, Santa Cruz Biotechnology). The sections were rinsed with IHC-Tek Washing Buffer for 2×5 min and subsequently incubated with secondary antibody (dilution of 1:200) for 1 h. For the detection, IHC-Tek DAB Peroxidase substrate kit containing DAB chromogen concentrate (#IW-1600, IHC world, Woodstock, MD, USA) was used. The slides were then incubated in IHC-Tek Mayer's Hematoxylin Solution (IW-1400, IHC world, Woodstock, MD, USA) for 10 min.

The slides were dehydrated in 95 % and 100% ethanol and followed by the clear xylene. Finally, the slides were air dried and added the coverslip with permanent mounting medium (#E01-18, Golden bridge international labs, Bothell, WA, USA).

2. Supplementary results

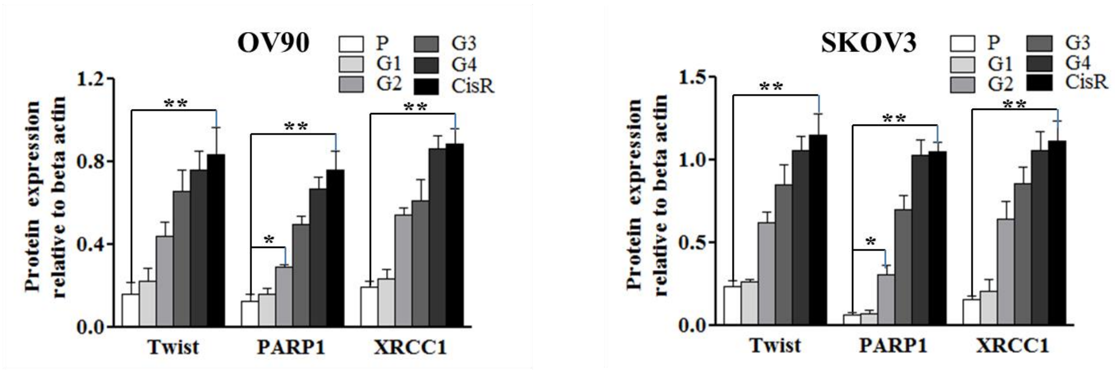

Figure S1. The expression level of Twist, PARP1 and XRCC1 during the development of cisplatin resistance in OC cells. P: Parental cell; CisR: Cisplatin resistant cells; G: Generation sublines. Values were represented as mean ± SD. *p < 0.05, **p < 0.01, compared with their respective parental cells.

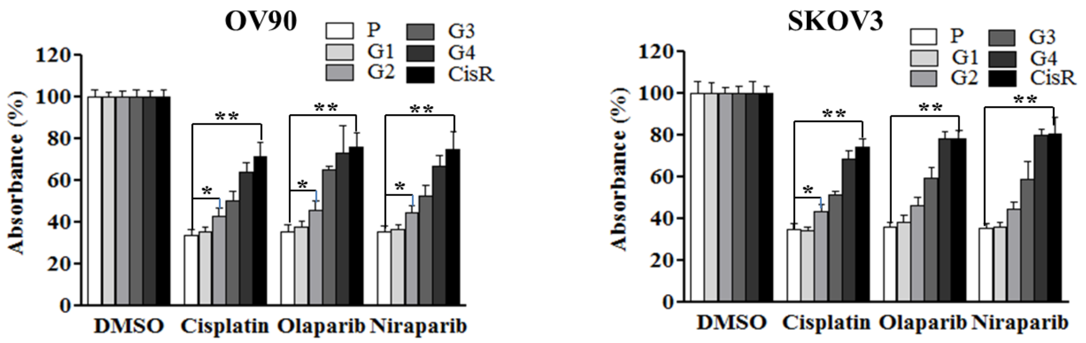

Figure S2. The cell growth rate measured as percentage of absorbance at 490 nm in OC cells. P: Parental cell; CisR: Cisplatin resistant cells; G: Generation sublines. Values were represented as mean ± SD. *p < 0.05, **p < 0.01, compared with their respective parental cells.

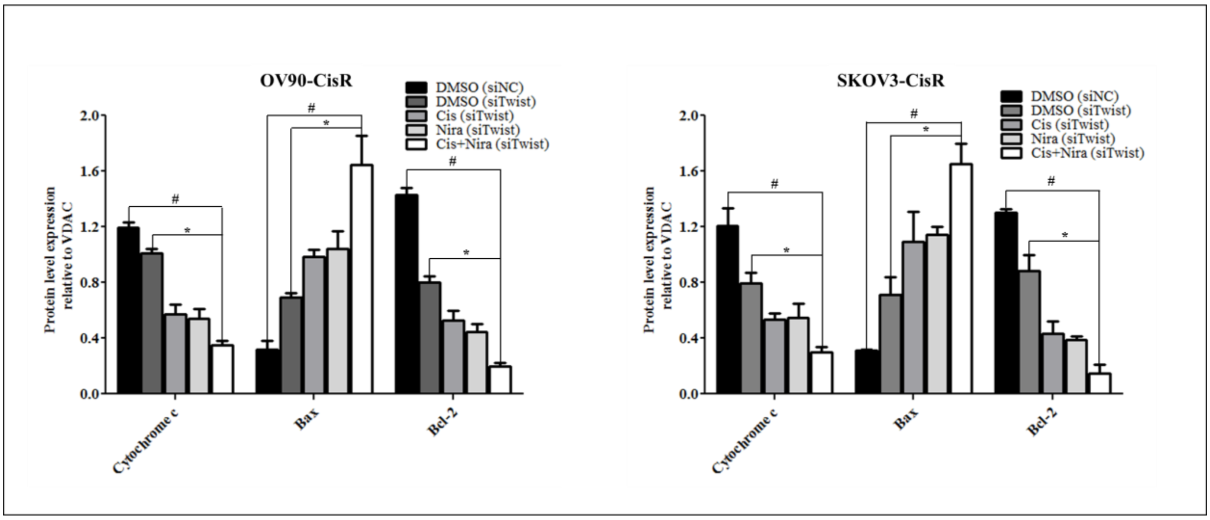

Figure S3. The expression levels of cytochrome *c*, Bax and Bcl-2 in mitochondrial fraction after drug treatments. Values were represented as mean ± SD. CisR: cisplatin resistant; siRNA: small interfering RNA; siNC: cisplatin resistant cells transfected with non-targeting negative control, siRNA; siTwist: cisplatin resistant cells transfected with Twist siRNA; DMSO: dimethyl sulfoxide, Cis: cisplatin; Nira; niraparib. Values were represented as mean ± SD. #p < 0.05, compared with siNC group; *p < 0.05, compared with the siTwist (DMSO) group.

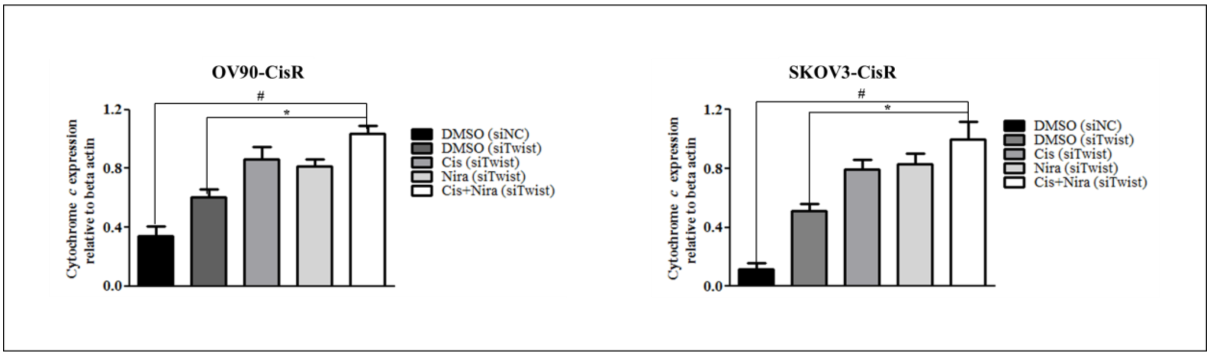

Figure S4. The expression levels of Cytochrome *c* in cytosolic fraction after drug treatments. Values were represented as mean ± SD. CisR: cisplatin resistant; siRNA: small interfering RNA; siNC: cisplatin resistant cells transfected with non-targeting negative control, siRNA; siTwist: cisplatin resistant cells transfected with Twist siRNA; DMSO: dimethyl sulfoxide, Cis: cisplatin; Nira; niraparib. Values were represented as mean ± SD. #p < 0.05, compared with siNC group; *p < 0.05, compared with the siTwist (DMSO) group.

3. Supplementary table

Table S1: The IC_{50} value of the three combination ratio in parental OC cells and siNC and Twist knockdown CisR OC cells

Groups	IC_{50} (μM)					
	OV90-P	SKOV3-P	OV90-CisR (siNC)	SKOV3-CisR (siNC)	OV90-CisR (siTwist)	SKOV3-CisR (siTwist)
Cis	19.38±0.79	18.45±1.01	80.93±2.59	102.80±5.86	51.96±2.60	65.05±7.62
Nira	31.46±2.91	28.31±1.14	98.70±6.13	87.36±4.23	57.10±2.05	44.84±3.62
Cis:Nira (1:1)	31.02±0.86	23.83±0.42	82.37±5.50	89.83±3.96	13.91±0.62	26.39±0.33
Cis:Nira (2:1)	28.85±0.35	21.88±0.35	82.36±3.59	98.02±5.97	27.86±1.24	49.20±1.33
Cis:Nira (1:2)	33.43±1.69	29.31±0.40	92.32±2.51	90.45±5.57	32.55±0.14	43.72±2.02

P: parental CisR: cisplatin resistant; siTwist: Twist siRNA; Cis: cisplatin; Nira: niraparib

Table S2: The dose (IC_{25}) of drugs and their respective CI value in Twist knockdown CisR OC cells

Cell lines	Drugs	IC_{25} (μM)	CI
OV90-CisR (siTwist)	Cis	18	0.39
	Nira	17.5	
SKOV3-CisR (siTwist)	Cis	21	0.42
	Nira	15	

CisR: cisplatin resistant; siTwist: Twist siRNA; Cis: cisplatin; Nira: niraparib
